# Prevalence and co-occurrence of compulsive buying, problematic Internet and mobile phone use in college students in Yantai, China: relevance of self-traits

**DOI:** 10.1186/s12889-016-3884-1

**Published:** 2016-12-01

**Authors:** Zhaocai Jiang, Mingyan Shi

**Affiliations:** Department of Psychology, School of Educational Science, Ludong University, Hongqi Middle Road 186, Zhifu District, Yantai, 264025 China

**Keywords:** Compulsive buying, Problematic Internet use, Problematic mobile phone use, Self-control, Self-esteem, Self-efficacy

## Abstract

**Background:**

Until now, most research in the prevalence of compulsive buying (CB) has been developed from samples in western developed countries, this study aimed to estimate the prevalence and co-morbidities of CB, problematic Internet use (PIU) and problematic mobile phone use (PMPU) in college students in Yantai, China. Moreover, based on the lack of research focusing on differences between CB and addiction, we will explore whether CB and PIU/PMPU individuals are characterized by the same self-traits (i. e., self-control, self-esteem and self-efficacy) related profile.

**Methods:**

A total of 601 college students were involved in this cross-sectional study. Compulsive buying, problematic Internet and mobile phone use and self-traits were assessed by self-reported questionnaires. The demographic information and use characteristics were included in the questionnaires.

**Results:**

The incidence of CB, PIU and PMPU were 5.99, 27.8 and 8.99% respectively. In addition, compared with rural students, students from cities are more likely to get involved in CB. Students using mobile phone to surf the Internet displayed higher risk of PIU than counterparts using computer. Students using Internet or mobile phone longer are more prone to problematic use. Furthermore, we found the strong correlations and high co-morbidities of CB, PIU and PMPU and self-control was the most significant predictor for all three disorders. However, self-esteem and self-efficacy were significant predictors only for CB.

**Conclusions:**

Our findings indicated that with the prevalence of CB and PMPU roughly equivalent to that demonstrated in previous studies, PIU in Chinese college students is serious and deserves more attention. Furthermore, besides the impulsive aspect common with addiction, CB is also driven by painful self-awareness derived from low self-regard which implies the obsessive-compulsive aspect.

## Background

Compulsive buying (CB) has been defined as a chronic and excessive form of shopping and spending characterized by intrusive thoughts and uncontrollable urges to buy that lead to repetitive purchasing episodes [1]. In estimating its prevalence, epidemiological surveys have confirmed percentages were about 4.9% with great variability ranging from 3.6 to 31.9% [2, 3], and a slightly higher prevalence (about 8.3%) was observed among university students [4]. Previous studies have indicated that the socio-cultural context and economic development might be critical factors influencing CB [4, 5]. Although several recent studies have investigated CB behavior with Chinese samples in Hong Kong and Macau [6], Taiwan [7] as well as China mainland [8], almost the entirety of knowledge in this area has been developed from samples in western developed countries, for example the United States, Germany, etc. [9, 10]. The only study investigating CB behavior in China mainland, a rapid developing emerging economy, included college students in Fuzhou and Chongqing in southern China [8]. Since different locations along with distinct consumer culture may affect CB behaviors, thus, in the present study we will estimate the prevalence of CB in Yantai, in eastern China.

In the last few years, an increasing number of behavioral addictions involving a great variety of behaviors and activities (such as work, sex, eating, gambling, etc.) have been identified [11, 12]. Among them, problematic Internet use (PIU) refers to an individual’s inability to control their Internet use, which in turn leads to feelings of distress and functional impairment of daily activities [13]. Actually, a large number of studies have estimated the prevalence of PIU in China and reported the incidence of PIU among Chinese adolescents is about 2.4–10.6% [14–16]. Along with the rapid development of smart phone, mobile phone is gradually completing many of the same tasks as an Internet connected computer. As estimated, by the end of 2015, the number of mobile phone users in China has reached 13.1 billion, and young adults (age 18–22) are the largest and fastest-growing group [17]. Moreover, the portability feature of mobile phone seems to make it an important way for students to regulate their negative emotions [18]. Thus, problematic mobile phone use (PMPU), a behavioral addiction analogous to PIU, has gained increasing attention in recent years, especially among youth in China and the incidence of PMPU among Chinese adolescents is about 4–10.6% [19].

Many studies have suggested similarities exist between CB and addiction in terms of the clinical characteristics [20, 21]. Moreover, high prevalence of substance use disorders in CB was found at rates ranging from 21 to 53% [22, 23]. Compulsive buyers also have a stronger motivation to buy on the Internet than at retail stores and connect to online shopping sites longer and more frequently [24]. However, there is always an ongoing debate whether CB is an addiction or obsessive-compulsive disorder [22, 25]. To explore the nature of CB and addiction, past studies have investigated the role of self-traits, such as self-esteem, self-efficacy and self-control, in these disorders. Self-control pertains to an individual’s capacity to resist inner desires so that he or she can achieve a more optimal outcome [26]. A large number of studies have demonstrated impaired self-control and rash impulsivity are associated with CB [27, 28], PIU [29, 30] as well as PMPU [30, 31], implying that they are all primarily driven by impulsivity and have been considered as the spectrum of impulse control disorders (ICD) [32]. On the other hand, self-esteem refers to an individual’s self-appraisal that involves either favourable or unfavourable attitudes [33]. Self-efficacy involves one’s self-judgement of his or her own capacity for accomplishing a given task [34]. Although little research has focused on the association between self-efficacy and CB, many studies have demonstrated people who display CB symptoms are also identified as having low self-esteem [35, 36] and CB may act as a coping response to one’s feelings of inadequacy. However, results on the relationships between self-esteem/self-efficacy and addiction-like symptoms seemed to be not entirely consistent. Some studies have shown an association between internet addiction and low levels of self-esteem and self-efficacy [37–40], but recent studies did not find the connections between self-esteem/self-efficacy and addiction-like symptoms, such as problematic Internet use and problematic mobile phone use [30, 31]. In view of this lack of agreement across studies, there appears to be an urgent and necessary need to advance in the identification of self-traits related profile for CB and PIU/PMPU.

At present, most of the research is concerned with the similar factors underlying CB and addiction [20, 21], however, research focusing on differences between them is rare. Thus, the goal of the present study was threefold: (1) to investigate the prevalence of CB/PIU/PMPU symptoms and possible demographic factors in a sample of Chinese college students; (2) furthermore, to determine the co-morbidities and associations among CB/PIU/PMPU symptoms; (3) to investigate whether CB and PIU/PMPU individuals are characterized by the same self-traits related profile.

## Methods

### Participants

Between June 2015 and January 2016, a cross-sectional study including 630 undergraduate students was conducted in Yantai, located in Shandong Province in eastern China. Out of the five universities in Yantai, three were selected at random (all ranked 200–300 in Chinese universities): Ludong University (n = 244), Yantai University (n = 189) and Shandong Technology and Business University (n = 197). Samples were randomly invited through campus advertisement with the purpose of this study. All subjects gave their informed consent for inclusion before they participated in the study. The survey was approved and supervised by the Institutional Review Board, sponsored by the China Association for Science and Technology (CAST) and the Ministry of Health of the People’s Republic of China. Questionnaires were administered to the participants in a classroom setting by a team of trained graduate students. 29 had to be excluded for not replying properly to all questionnaires, so the final sample consisted of 601 participants. All students ranged in age from 18 to 24 years (M ± SD = 20.63 ± 1.52).

### Measures

#### Demographic information and use characteristics

Participants reported the following demographic information: age, gender, family background (“city”/“rural”) and whether they were the only child in the family (“yes”/“no”). For use characteristics, participants answered the following questions: time spent per day (TPD) on shopping/Internet/mobile phone, Internet or mobile phone use history (UH), the most common way of shopping (retail store, computer or mobile phone) and the most common way of surfing the Internet (computer or mobile phone).

### Compulsive Buying Scale (CBS)

CB was measured by the Compulsive Buying Scale which consists of 7 items including characteristic aspects of CB, such as the preoccupation with buying, misuse of credit cards, malaise when not shopping, a lack of control over buying and frequent shopping and buying to feel better [1]. The original scale was firstly translated into Chinese by 2 graduate students and modified by researchers. To ensure no ambiguity, the Chinese version of this scale was back-translated into English by foreign teachers whose native language is English and second language is Chinese. After several rounds of discussion and modification, the Chinese version of CBS was adopted in the present study. A lower score is associated with higher level of CB, whereby a cut-off score equal to −1.34 or lower indicates having CB. The Cronbach’s alpha coefficient of CBS in the present study was 0.78.

### Problematic Internet Use Diagnostic Questionnaire (DQ)

The translation process of DQ was similar as CBS. The DQ comprised eight items [13]. “Yes” was scored 2 point, and “No” was scored 1 points. Respondents who scored 13 or higher were classified as addicted Internet users. Previous study indicated the criterion-related validity value of DQ was 0.72 and coefficient alpha was 0.87 [14]. The coefficient alpha in the present study was 0.81.

### Problematic Mobile Phone Use Scale (PMPUS)

The PMPUS is a 16-item scale developed based on Young’s (1998) Problematic Internet Use Scale [13, 41]. It consists of four subscales: withdrawal symptoms, salience, social comfort and mood changes. Higher score on this measure indicates greater level of mobile phone abuse. Both exploratory and confirmatory factor analyses supported the construct validity of the four subscales [41]. In this study the Cronbach’s alpha coefficient is 0.86.

### Self-control Scale (SCS)

We employed a Chinese version of SCS revised from Tangney’s (2004) original version and contained 19 items [26, 42]. Participants assessed each item from 1 (not at all like me) to 5 (very much like me). Higher scores on this scale indicate stronger capability for self-control and greater likelihood of attaining goals. The SCS has strong internal consistency (α = 0.86) and good test–retest reliability (r = 0.89) [42]. In our study, the Cronbach’s alpha coefficient is 0.80.

### Self-esteem scale

Self-esteem was assessed by the Chinese version of Rosenberg Self-esteem Scale [33, 43]. This scale includes ten items that evaluate people’s positive and negative feelings about the self. Higher values represented higher self-esteem. It has good reliability and validity in Chinese adolescents [43]. Cronbach’s α of the present sample was 0.83.

### General self-efficacy scale

Self-efficacy was assessed using the General Self-efficacy Scale first developed by Schwarzer and was translated into Chinese by Zhang [44]. It is a 10-item 4-point Likert scale. Higher numbers demonstrate higher efficacy beliefs. Cronbach’s alpha of the scale varied from 0.75 to 0.91 [44] and for the present sample was 0.88.

### Data analysis

Analyses were performed with SPSS 17.0. The demographics and use characteristics of CB, PIU and PMPU were analyzed by chi-squared. The relationships between levels of CB, PIU and PMPU were explored using Pearson’s correlation coefficient. Logistic regression analyses were performed to examine the predictive effects of self-traits for CB, PIU and PMPU after controlling for gender, family background and family structure.

## Results

**Fig. 1:**
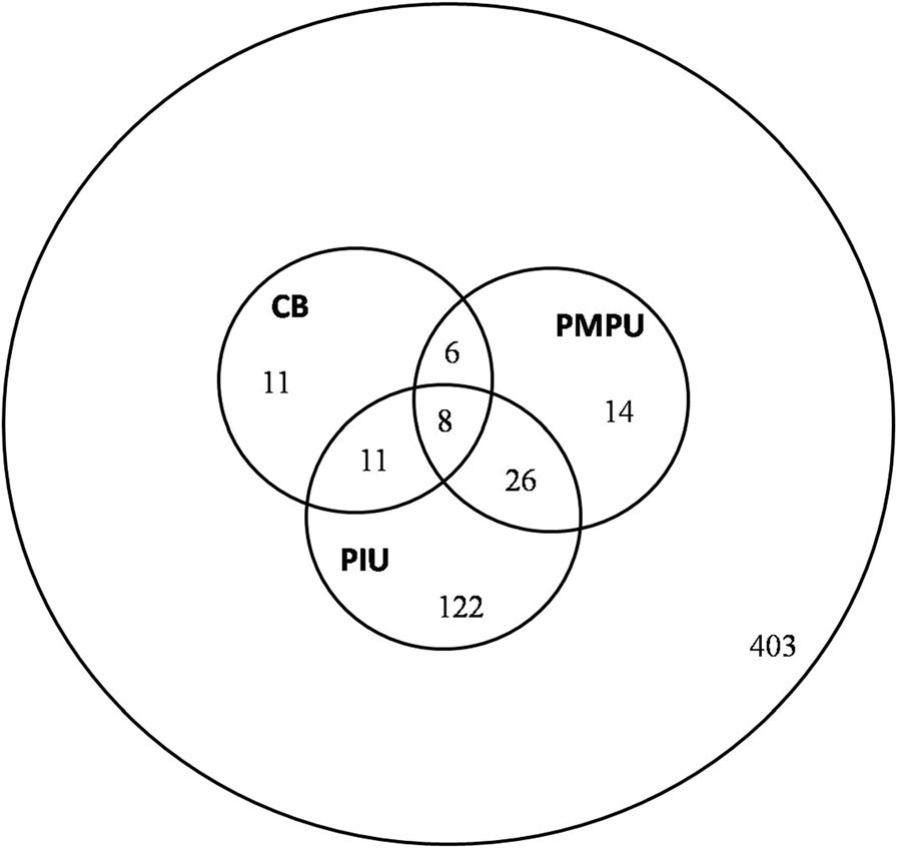
Prevalence and co-occurrence of CB, PIU and PMPU. Figures in the circles show the number of participants in the corresponding category

Figure 1 illustrates the prevalence and co-morbidities of CB, PIU and PMPU. 67.1% of participants (n = 403) showed none of the 3 behavioral disorders tested in our study. Participants classified as CB, PIU and PMPU were 36 (5.99%), 167 (27.8%) and 54 (8.99%) respectively. Moreover, participants displaying co-occurrence of CB and PMPU, CB and PIU, PMPU and PIU were 6 (0.10%), 11(1.83%), 26 (4.33%) respectively. 1.33% (n = 8) of the participants presented with co-occurrence of 3 behavioral disorders.

Table 1 shows the correlation coefficients of CBS, PIU and PMPU. Higher scores on PIU and PMPU indicate greater levels of overuse and the result showed PIU and PMPU were positively correlated (r = 0.52, *p* < 0.01). CBS scores were negatively correlated with PIU (r = −0.28, *p* < 0.01) and PMPU (r = −0.43, *p* < 0.01). For higher CBS score is associated with lower level of CB, thus these results indicated that the levels of CB, PIU and PMPU were positively correlated with each other in our sample.

**Table 1:** Pearson correlations between CBS, PIU and PMPU

Variables	1.CBS	2.PIU	3.PMPU
1	_		
2	-.28^**^	_	
3	-.43^**^	.52^**^	_

Note: ^**^ *p* < 0.01

Demographics and use characteristics of behavior disorders are illustrated in Table 2. Compared with rural students, students from cities had a higher risk of CB (χ^2^ = 3.90, *p* < 0.05). However, gender and family structure had no significant influence on any of the 3 behavior disorders. As for use characteristics, students spending more time on buying, Internet or mobile phone per day were more likely to engage in corresponding problematic behaviors (for CB: χ^2^ = 34.3, *p* < 0.001; for PIU: χ^2^ = 24.7, *p* < 0.001; for PMPU: χ^2^ = 26.1, *p* < 0.001). The earlier students were exposed to the Internet or mobile phone, the more likely they were to problematically use it (for PIU: χ^2^ = 8.70, *p* < 0.05; χ^2^ = 7.90, *p* < 0.05). In addition, we observed students using mobile phone to surf the Internet displayed higher risk of PIU than counterparts using computer (χ^2^ = 6.60, *p* < 0.05). However, participants adopting differing ways of shopping presented no significant difference in the possibilities of becoming compulsive buyers (χ^2^ = 0.27, *p* > 0.05).

**Table 2:** Demographics and use characteristics of behavior disorders

		CB			PIU			PMPU		
		Yes	No	χ^2^	Yes	No	χ^2^	Yes	No	χ^2^
Gender	Male	18	264		82	200		25	257	
	Female	18	301	0.15	85	234	0.44	29	290	0.01
Family background	City	20	220		72	168		24	216	
	Rural	16	345	3.90^*^	95	266	0.98	30	331	0.50
Only child or not	Yes	10	200		61	149		23	187	
	No	26	365	0.87	106	285	0.26	31	360	1.53
TPD	<1 h	2	114		8	39		1	28	
	1–2	15	302		20	83		1	84	
	2–3	9	121		32	124		9	135	
	3–4	6	20		30	72		5	103	
	>4 h	4	8	34.3^***^	77	116	24.7^***^	38	197	26.1^***^
UH	<3y	_	_		21	101		3	17	
	3–5y	_	_		51	112		5	109	
	5–10y	_	_		74	175		21	251	
	>10y	_	_	_	21	46	8.70^*^	25	170	7.90^*^
Means	Retail store	15	211		_	_		_	_	
	Computer	1	16		42	157		_	_	
	Mobile phone	20	338	0.27	125	277	6.60^*^	_	_	_

Note: *TPD* time spent per day, *UH* use history

^*^ *p* < 0.05, ^***^ *p* < 0.001

Results of the logistic regression analysis which included the CB or addiction status (0 = Non- CB/PIU/PMPU, 1 = CB/PIU/PMPU) as the criterion variable are presented in Table 3. All three self-traits were found to be significant predictors for CB. However, only self-control was found to be significantly associated with Internet and mobile phone use addiction, we did not observe the predictive effects of self-esteem and self-efficacy for addictive behaviors.

**Table 3:** The regression analysis of self-traits for CB, PIU and PMPU

	B	Wald	*p*	OR	95% CI
CB *Nagelkerke’s R* ^*2*^ *= 0.329, p < .001.*					
Self-control	-.101	27.746	<.001	.903	.869–.938
Self-esteem	-.073	4.021	.041	.956	.859–1.064
Self-efficacy	-.085	4.853	.034	1.101	.980–1.234
PIU *Nagelkerke’s R* ^*2*^ *= 0.206, p < .001.*					
Self-control	-.105	32.863	<.001	.905	.883–.928
Self-esteem	-.032	1.050	.305	.969	.911–1.030
Self-efficacy	-.006	.038	.845	.994	.932–1.060
PMPU *Nagelkerke’s R* ^*2*^ *= 0.221, p < .001.*					
Self-control	-.107	37.961	<.001	.899	.869–.930
Self-esteem	.007	.021	.884	1.007	.920–1.101
Self-efficacy	.021	.174	.677	1.021	.927–1.124

## Discussion

In the present sample, participants classified as CB and PMPU were 5.99 and 8.99% respectively, roughly equivalent to previous studies estimated [4, 18, 19]. However, it is worth noting that the incidence of PIU in this study was 27.8%. Although the prevalence rates of PIU seem to vary due to differences in samples, screening measurements, social and cultural context [14, 32], even after taking these differences into consideration, our results still indicate that PIU among Chinese university students is serious and seems to be enhanced compared with previous investigations in China [14]. One of the main reasons is the rapid expansion of the Internet and the increased substantial exposure of university students to the Internet through mobile phone and other devices in recent years. Furthermore, we found that students using mobile phone to surf the Internet displayed higher risk of PIU than counterparts using computer. At present, in Chinese college students surfing the Internet with computers is mostly used to accomplish a specific task (such as work, learning, etc.) which brings limited pleasure. However, due to the portability of mobile phone, the mobile network is often used to kill time, shopping or entertainment which is usually accompanied by more enjoyment and more easily leads to addiction [45]. These combined findings deserve more attention for they indicate that although excessive use of the Internet and mobile phone both function as coping with underlying negative affective states [18, 20, 21], the emergence of mobile phone does not replace or reduce, but instead seems to aggravate the incidence of PIU. Moreover, more specific subtypes of PMPU need to be identified in the future because our results suggest that in part, PMPU may actually imply students’ inability to control surfing the Internet by mobile phone.

In relation to demographic determinants, our results suggested that gender or family structure did not establish significant differences between those with and without CB/PIU/PMPU. Actually, regarding gender, some research has detected significantly higher possibility of females becoming compulsive buyers in the general population samples [5, 46] and higher possibility of male students involved in PIU in Chinese adolescents [14–16]. Although our results are more in line with other studies indicating no significant gender differences in CB [9, 10] and PIU [47, 48], considering only college students from one single city are included in our sample, it should be cautious when our conclusions are generalized to other populations in China. As for family background, we found students from cities are more likely to become CB than their rural counterparts. Since city family background generally represents higher family social economic status and income level relative to rural in China, our finding may suggest that CB of university students is related to higher family income, although this needs to be further verified. In addition, consistent with the fact that CB or addiction is typified by intense preoccupation and involves a lot of time engaging in this behavior [11], we found the more time participants tend to spend on shopping/Internet/mobile phone per day, the more likely they will get involved in CB/PIU/PMPU. Furthermore, in line with previous studies [14, 49], we showed that participants using Internet or mobile phone longer display higher risk of problematic use. This is not surprising when we consider problematic Internet or mobile phone use comparable to substance addiction in which dependence-related symptoms are reinforced with time for drug tolerance [50, 51].

In accordance with previous findings on the close relations between CB, PIU and PMPU [20–24], in the present study we observed the strong correlations and high co-morbidities of the 3 behavioral disorders. Moreover, our findings showed self-control was the most significant predictor for all 3 disorders, which is in line with previous studies demonstrating lack of effort control in these disorders and provides additional evidence for common impulsive aspect underlying CB and addiction [27–32]. Many studies have shown that compared with healthy controls CB patients or addicts are both more likely to experience negative affect states [5, 18]. Individuals with deficit in self-control are prone to act impulsively, without analytical or deliberative processing in emotional contexts. This sense of urgency probably compels individuals with behavioral addictions or CB to alleviate negative emotions with maladaptive actions [52]. On the other hand, the maladaptive behaviors performed in emotional contexts often result in negative outcomes (personal, professional, social) [1, 14, 41], which in turn promote the experience of negative emotions. Thus, for low self-controllers CB or addictions may serve as a commonly used mechanism to cope with negative feelings. Given CB, PIU and PMPU are all characterized by lack of self-control, strengthening an individual’s self-control could be beneficial for the treatment of these symptoms. Actually, the literature on self-control has suggested that self-control can be trained [53, 54]. Therefore, more importance should be attached to self-control training for the clinical intervention of CB/PIU/PMPU in future studies.

In the present study we found self-esteem and self-efficacy could significantly predict CB, however the effects were not significant for PIU and PMPU. Distinct from PIU and PMPU, the combination of decreased self-esteem and self-efficacy implies low self-regard of CB individuals. In accordance with our findings, researchers have argued that CB serves the function of escaping chronic painful affect derived from low self-regard, motivating individuals to narrow their attention to immediate, concrete tasks (i.e., the act of buying) [55, 56]. Thus, for CB individuals buying can be regarded as an attempt to strengthen the identity and to bridge the discrepancy between one’s desired and actual self [56, 57]. Furthermore, the poor self-regard and its relative escape motive appear to characterize the obsessive-compulsive, rather than the impulsive aspect of compulsive buying [22, 25]. Therefore, our results indicate that beyond the impulsive aspect of PIU and PMPU, CB is an obsessive-compulsive and impulse control disorder.

There are several limitations of this study that need attention. Although this study was able to replicate previous research evidence, the relatively small sample size may warrant a conservative approach to interpretation. Moreover, only university students in Yantai, a medium developed city in eastern China, are recruited in our study. Future studies should use more representative samples that would allow results to be generalized more confidently. Additionally, all information was obtained from self-reported questionnaires with cross-sectional design. Self-reported questionnaire could result in the possibility of response bias. Cross-sectional design does not allow for causality relationships. Thus, in the future multiple assessments and longitudinal studies are required to provide a richer and more thorough understanding of these behavioral disorders.

## Conclusions

In summary, our results indicate that the prevalence of CB and PMPU were equivalent to that demonstrated in previous studies, but PIU in Chinese college students is serious and deserves more attention. Additionally, compared with rural students, students from cities are more likely to get involved in CB. Participants using mobile phone to surf the Internet display higher risk of PIU than counterparts using computer. Longer Internet or mobile phone use history is associated with higher risk of problematic use. Furthermore, we found impaired self-control was the common underlying mechanism which underpins high co-morbidities of CB, PIU and PMPU, while CB is separately driven by painful self-awareness derived from low self-regard which implies the obsessive-compulsive aspect. Thus, future studies for the clinical intervention of CB/PIU/PMPU should attach more importance to self-control training and ways of getting away from low self-regard should be further explored for CB individuals separately. Finally, if possible, knowledge of the different nature of CB and addiction as well as their relations with self-traits should be spread to the public and this might help those being troubled get rid of their disorders.

## Abbreviations

CB: Compulsive buying
PIU: Problematic Internet use
PMPU: Problematic mobile phone use
